# Association Analysis of *SLC11A1* Polymorphisms with Somatic Cell Score in Chinese Holstein Cows

**DOI:** 10.3390/ani15101370

**Published:** 2025-05-09

**Authors:** Kai Liu, Yufang Liu, Tuo Li, Qiuling Li, Jinyu Wang, Yongfu An, Yuze Yang, Kaiyang Li, Mingxing Chu

**Affiliations:** 1 State Key Laboratory of Animal Biotech Breeding, Institute of Animal Science, Chinese Academy of Agricultural Sciences, Beijing 100193, China; 2 College of Animal Science and Technology, Yangzhou University, Yangzhou 225009, China; 3 Hebei Key Laboratory of Animal Diversity, Langfang Key Laboratory of Cell Engineering and Application, College of Life Sciences, Langfang Normal University, Langfang 065000, China; 4 Hebei Animal Science and Veterinary Medicine Institute, Baoding 071000, China; 5 Beijing General Station of Animal Husbandry, Beijing 100101, China

**Keywords:** Holstein cows, mastitis resistance, candidate genes, SNP selection

## Abstract

Mastitis is an important disease limiting milk production in dairy cows. Somatic cell score is commonly used as one of the main ways to gauge the level of mastitis in dairy cows, with higher somatic cell scores usually indicating possible mastitis. However, the main molecular markers affecting somatic cell scores remain unknown. The aim of this study was to investigate the association between single nucleotide polymorphisms in the *SLC11A1* gene and somatic cell score in Chinese Holstein cows. In this study, 210 Chinese Holstein cows were genotyped and potential SNPs were detected by DNA sequencing, PCR-SSCP and PCR-RFLP analysis. Our results revealed two SNPs were identified in the CDS region of *SLC11A1*: c.723C>T and c.1144C>G. For the c.723C>T polymorphic site, two genotypes (*AA*, *AB*) were found and the genotype frequencies were 0.790 and 0.210, respectively. The results of the association analysis showed that the mean somatic cell score of the *AA* genotypes were significantly lower than those of the *AB* genotypes, suggesting that the *A* allele is a potential marker for improving mastitis resistance in Chinese Holstein cows. For the c.1144C>G polymorphic site, three genotypes (*CC*, *CD,* and *DD*) were found and the genotype frequencies were 0.629, 0.352 and 0.019, respectively. The association analysis revealed that the mean somatic cell score of *CC* genotypes was lower than that of *CD* and *DD* genotypes, however, no significant differences were observed among the various genotype groups when subjected to pair-wise comparisons. The bioinformatic analysis showed that these mutations affected the secondary and tertiary structure of *SLC11A1* mRNA, suggesting that they may affect gene expression or protein translation and function. Finally, we predicted the SLC11A1 protein interaction network and found that SPI1, NOD2, TLR2 and S100A12 interacted with SLC11A1 and were reported as candidate genes associated with mastitis resistance. The results indicated that the SNP (c.723C>T) could be potential molecular marker for improving mastitis resistance traits in Chinese Holstein cows. We recommend further validation of this SNP in larger populations and its potential integration into breeding programs to enhance mastitis resistance in dairy cows.

## Simple Summary

Milk production traits, including milk yield, fat content, and protein content, are critical economic indicators in the dairy industry. Somatic cell score (SCS) is another important metric, as it reflects the health status of dairy cows and impacts milk quality. The *SLC11A1* gene, which is involved in the transport of divalent metals such as iron and manganese, has been implicated in influencing immune responses and potentially milk production traits. Single nucleotide polymorphisms (SNPs) are variations at a single nucleotide position in the DNA sequence and play a vital role in genetic studies of milk production traits. In this research, we identified two SNPs within the *SLC11A1* gene and analyzed their associations with SCS. The results indicated that specific SNPs in the *SLC11A1* gene were significantly associated with SCS, suggesting that these genetic variations could influence the health status and milk quality of Chinese Holstein cows.

## 1. Introduction

Mastitis is the most common and costly disease on dairy farms, with adverse effects including reduced milk production, impaired fertility and premature culling [1,2]. Traditional breeding strategies for mastitis resistance based on phenotypic selection have not been very successful due to the low heritability of mastitis resistance traits [3]. An indirect selection strategy to reduce mastitis is based on milk somatic cell score (SCS), which is strongly and positively correlated with clinical mastitis [4,5]. Single nucleotide polymorphism (SNPs) is the third generation of genetic markers, which arealterations in the DNA sequence where one nucleotide is replaced by another at a specific genomic position, deviating from the reference genome. In livestock breeding, SNPs are important in determining an animal’s genotype and its relationship to production, reproductive and economic traits [6,7,8]. SNPs and genomic linkage technology have been used to identify genes for milk production traits in dairy cows [9]. The impact of mastitis can be reduced by genetic testing and indirect selection for cows with lower SCS [10,11]. Natural resistance-associated macrophage protein 1 (*NRAMP1*), also known as solute carrier 11A1 (*SLC11A1*), is a member of the solute carrier family 11 [12,13,14]. *SLC11A1* is a highly conserved gene and was first identified in mouse IC3 [15]. It is located on bovine chromosome 2q43-44, contains 16 introns and 15 exons, and *SLC11A1* encodes a multichannel membrane protein that functions by translocating divalent cations (iron and manganese) involved in iron metabolism and host resistance to specific pathogens [13]. Its role in resistance or susceptibility to bacterial infection makes it a promising candidate gene for breeding disease-resistant animals [16]. This gene plays an important role in innate immunity by preventing bacterial growth in macrophages during the early stages of infection [17]. Mutations in SLC11A1 have been found to confer susceptibility to a variety of intracellular pathogens, including Mycobacterium, Salmonella and Leishmania [18,19,20]. In addition, the *SLC11A1* gene has been found to be associated with natural resistance to brucellosis in cattle [21,22] and buffalo [23,24]. It is noteworthy that SNP in the *SLC11A1* gene was identified as a significant genetic marker and susceptibility factor for mastitis tolerance/susceptibility in both Holstein and Swiss Brown cows [25]. Furthermore, a mutation was identified at 1139bp in exon 11 of the *SLC11A1* gene, which was significantly associated with the development of clinical mastitis [26]. Furthermore, a comparison of the transcriptomes of circulating leukocytes from healthy cows with those from cows with naturally occurring subclinical or clinical mastitis revealed some differentially expressed genes, including *SLC11A1* [27]. These results suggest that *SLC11A1* is a candidate gene for disease resistance in cattle. Since *SLC11A1* is involved in innate immunity by killing bacteria, we hypothesized that it is a candidate gene for mastitis resistance.

In the present study, we analyzed the relationship between polymorphisms in *SLC11A1* and SCS in Chinese Holstein cows, and the potential biological functions of SNPs in this gene. The results may provide a theoretical basis for marker-assisted selection for mastitis resistance in Chinese Holstein cows.

## 2. Materials and Methods

### 2.1. Blood Sample Collection and DNA Preparation

The 210 Holstein cows involved in this study came from four dairy farms in Hebei Province, China (70 from Farm 1, 70 from Farm 2, 35 from Farm 3, and 35 from Farm 4). These cows are the progeny of five bulls with 39, 41, 42, 43 and 45 offspring respectively. The number of cows at three parities was 65 (first birth), 71 (second birth) and 74 (third birth), respectively. This study collected data on various factors related to dairy cows. These included recording of body condition scores on a scale of 1 to 5 in increments of 0.25 with 3 being the ideal score. In addition, milk yield data were collected daily from each cow throughout the lactation period. Stage of lactation: Days in milk (DIM) categorized as early (1–100 DIM), mid (101–200 DIM), and late (>200 DIM) per dairy management standards. Other relevant data such as health status and management practices were also recorded. The average SCC (somatic cell count) of the cows on the four farms was 700,000/mL, 550,000/mL, 400,000/mL and 450,000/mL. Blood was collected from the jugular vein of each cow in 10 mL samples, anticoagulated with ACD and stored at −20 °C. Genomic DNA was extracted from blood samples using the phenol-chloroform method, then dissolved in TE buffer (10 mmol/L Tris-HCl, 1 mmol/L EDTA) and stored at −20 °C.

Danish electronic counter (Series 300 Fossomatic Type 75200) was used to determine SCC of 210 Holstein cows’ milk once per month, for a maximum of 10 times each cow. The average of these counts was the SCC of the parity. Hypothesis testing requires that errors are normally distributed and subgroup variances are homogeneous in analysis of variance. Therefore, the SCC of abnormal distribution characteristic must be converted to SCC of normal distribution before the analysis. Ali and Shook [28] reported that the converted SCC data have widely normal distribution and homogeneity characteristic, the logarithm conversion of SCC is the best way to obtain normal distribution. Somatic cell score (SCS = log_2_^(SCC/100)^ + 3, the unit of SCC is 1000 cells/mL) has the normal distribution characteristic, has been the criterion measure of SCC of the National Cooperative Dairy Herd Improvement Program of USA. The number, father’s number, farm, parity and SCS of each cow were recorded.

### 2.2. Primer Sequences and PCR Amplification

Primers were designed using the bovine *SLC11A1* mRNA sequence. Primer sequences, target fragment lengths and annealing temperatures are listed in Table 1. The polymerase chain reaction (PCR) was performed in a 25 μL volume containing approximately 2.5 μL 10× PCR buffer (50 mmol/L KCl, 10 mmol/L Tris-HCl (pH 8.0), 0.1% Triton X-100), 1.5 μL MgCl_2_ (1.5 mM), 2.0 μL dNTPs (2.5 mM each), 0.5 μL each forward and reverse primers (20 μM), 50 ng genomic DNA and 1 U Taq DNA polymerase (SABC, Beijing, China). PCR conditions were as follows: pre-denaturation at 94 °C for 6 min; followed by 32 cycles of denaturation at 94 °C for 30 s, annealing for 30 s, extension at 72 °C for 30 s; with a final extension at 72 °C for 10 min, all operations were performed on a Mastercycler^®^ 5333 (Eppendorf AG, Hamburg, Germany).

### 2.3. Single Strand Conformation Polymorphism (SSCP) Detection

A volume of 3 μL PCR product was transferred to an Eppendorf tube and mixed with 7 μL gel loading solution containing 98% formamide, 0.025% bromophenol blue, 0.025% xylene cyanol, 20 mmol/L EDTA (pH 8.0), 10% glycerol. The mixture was agitated and denatured at 98 °C for 10 min, rapidly placed on ice for 7 min and loaded onto 10–12% neutral polyacrylamide gels (acrylamide:bisacrylamide = 29:1). Electrophoresis was performed in 1× Tris borate (pH 8.3)-EDTA buffer at 120 V for 15–20 h at 4 °C. After electrophoresis, the DNA fragments in the gels were visualized by silver nitrate staining, photographed and analyzed using an AlphaImager^TM^ 2200 and 1220 Documentation and Analysis System (Alpha Innotech Corporation, San Leandro, CA, USA).

### 2.4. Cloning and Sequencing

Amplification products from P1-P16 were analyzed by SSCP. Each DNA fragment was inserted into the pGEM-T Easy vector (Promega) according to the manufacturer’s instructions at 16 °C overnight. Ligation reactions were performed in 10 μL volumes containing PCR product 1 μL, pGEM-T Easy vector (50 ng/μL) 1 μL, T4 ligase (3 U/μL) 1 μL, 2× ligation buffer 5 μL, ddH_2_O 2 μL. The recombinant plasmid was transformed into competent *Escherichia coli* DH5α. Positive clones were identified by restriction enzyme digestion. Five clones of each genotype were selected for sequencing. Each clone was sequenced twice on an ABI3730 automated sequencer (Perkin Elmer Applied Biosystems, Foster City, CA, USA) by Shanghai Invitrogen Biotechnology Ltd. Co. (Shanghai, China).

### 2.5. Restriction Fragment Length Polymorphism (RFLP) Analysis

Two mutations (c.723C>T and c. 1144C>G) were detected in *SLC11A1* by sequencing. Based on the sequences, we found two restriction enzymes to carry out RFLP analysis. Restriction enzyme reaction was carried out in 15 μL volume by mixing 5 μL of PCR products, 5 U *Bpu*10I (New England Biolabs, Beverly, MA, USA) or 5 U *PstI* (New England Biolabs, Beverly, MA, USA) and 1 μL corresponding 10× reaction buffer and incubating at 37 °C overnight. The resulting fragments were separated by electrophoresis on 2% agarose gel (Promega) in parallel with a 100 bp DNA marker.

### 2.6. Bioinformatics Analysis

RNAFOLD 2.6.3 (http://rna.tbi.univie.ac.at//cgi-bin/RNAWebSuite/RNAfold.cgi (accessed on 22 February 2024)) was used to predict the mRNA secondary structure of wild-type and mutated *SLC11A1*. The tertiary structure of the target proteins was analyzed using SWISS-MODEL software [29] (https://swissmodel.expasy.org/ (accessed on 22 February 2024)). STRING 11.0 software (https://string-db.org/cgi/input.pl (accessed on 23 February 2024)) was used to analyze SLC11A1 proteins interactions.

### 2.7. Statistical Analysis

The following statistical model was fitted to compare difference of SCS among *SLC11A1* genotypes.

y_ijklm_ = μ+ S_i_ + H_j_ + P_k_ + G_l_ + e_ijklm_

where *y_ijklm_* is SCS, *μ* is population mean, *S_i_* is the fixed effect of the *i*th bull (*i* = 1, 2, 3, 4, 5), *H_j_* is the fixed effect of the *j*th dairy farm, *P_k_* is the fixed effect of the *k*th birth (*k* = 1, 2, 3), *G_l_* is the fixed effect of the *l*th genotype, *e_ijklm_* is the random error effect of each observation. Bull Effect (S_i_): The inclusion of the bull effect accounts for the genetic relatedness among the cows, as they are progeny of five different bulls. This ensures that the genetic background of the cows is considered in the analysis. Farm Effect (H_j_): The farm effect adjusts for environmental and management differences across the four dairy farms, ensuring that farm-specific factors do not confound the results. Parity Effect (P_k_): The parity effect accounts for differences in SCS due to the lactation stage of the cows, as SCS can vary with parity. Genotype Effect (G_l_): This is the primary factor of interest, representing the association between SLC11A1 genotypes and SCS. eijklm: The random error term captures unexplained variability in SCS values, ensuring that the model accounts for individual differences not explained by the fixed effects. Calculations were achieved using Proc GLM (General Linear Model) of SAS (V.8.12). Mean separation procedures were performed using a least significant difference test.

## 3. Results

### 3.1. SSCP Detection and Genotyping

**Figure 1:**
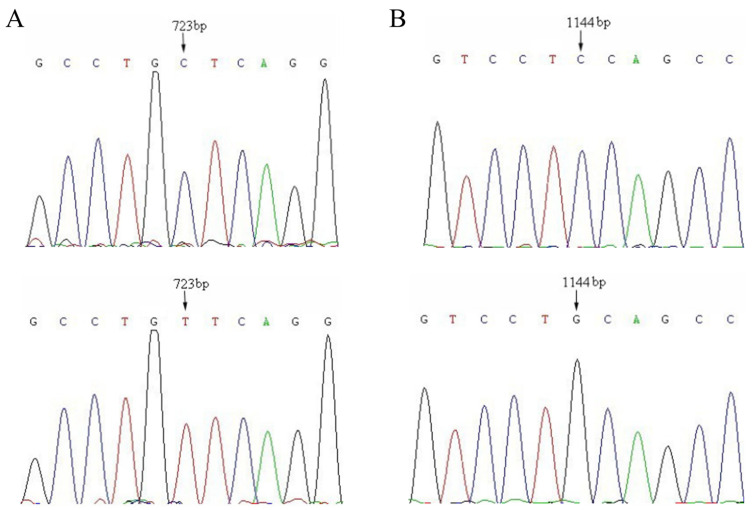
Two SNPs of *SLC11A1* in Chinese Holstein cows for primers P8 (**A**) and P11 (**B**).

SSCP genotypes were identified by mobility shift due to conformational difference of the single-stranded DNAs of the amplified fragments by each primer, which is caused by nucleotide variation. The results showed polymorphisms only in P8 and P11 in the CDS in *SLC11A1* gene. Sequence alignment between different genotypes of primer P8 revealed a C to T mutation at the 723 locus (exon 8) according to the mRNA sequence of the bovine *SLC11A1* (GenBank accession number NM_174652) of exon 8 (Figure 1), and this mutation caused an amino acid change (Ala to Val) at residue 217 (A217V). Sequence alignment between different genotypes of primer P11 revealed a C to G mutation at position 1144 according to the mRNA sequence of bovine *SLC11A1* (GenBank accession number NM_174652) (Figure 1), and c.1144C>G caused an amino acid change (Pro to Ala) at residue 356 (P356A). The C to T transition at site 723 and the C to G transversion at site 1144 changed the recognition site of the restriction endonuclease *Bpu10I* and *PstI*, respectively, which could be detected by PCR-RFLP. Both mutation sites are missense mutations.

### 3.2. Bpu10I Analysis of the c.723C>T of SLC11A1

**Figure 2:**
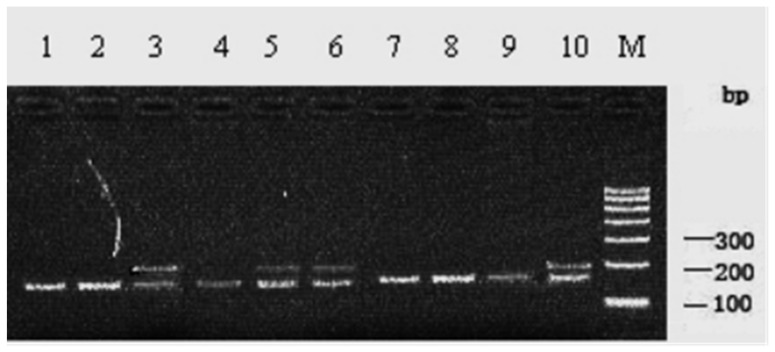
*Bpu10I*-RFLP patterns of PCR products of c.723C>T site (primer P8) in 2% agarose gel. 1, 2, 4, 7, 8, 9: AA genotype; 3, 5, 6, 10: AB genotype; M. DNA marker I.

The 199 bp PCR product with primer P8 was digested completely with the restriction endonuclease *Bpu10I* and genetic polymorphisms were detected by PCR-RFLP technique. Two genotypes were identified in 210 Chinese Holstein cows, which are *AA* (199 bp) and *AB* (42 bp/157 bp/199 bp) (Figure 2).

### 3.3. PstI Analysis of the c.1144C>G of SLC11A1

**Figure 3:**
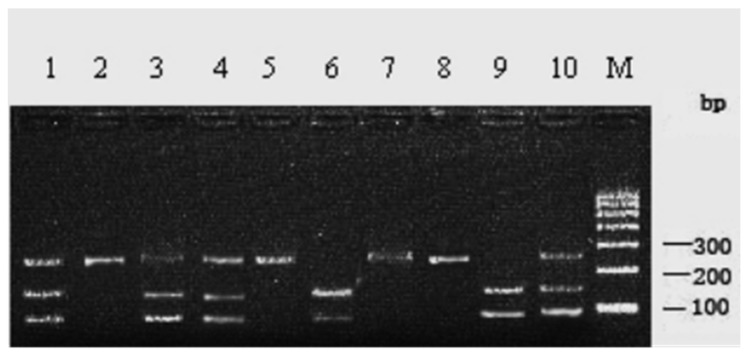
*PstI*-RFLP patterns of PCR products of c.1144C>G site (primer P11) in 2% agarose gel. 2, 5, 7, 8: CC genotype; 1, 3, 4, 10: CD genotype; 6, 9: DD genotype; M. DNA marker I.

The 243 bp PCR product with primer P11 was digested completely with the restriction endonuclease *PstI* and genetic polymorphisms were investigated by PCR-RFLP. Three genotypes (*CC*, 243 bp; *CD*, 95 bp/148 bp/243 bp; *DD*, 95 bp/148 bp) were detected in 210 Chinese Holstein cows (Figure 3).

### 3.4. Genetic Characteristics of Polymorphic Sites of SLC11A1 Gene in Chinese Holstein Cows

It was shown that *AA* and *CC* were the dominant genes at the c.723C>T and c.1144C>G loci, with genotype frequencies of 0.790 and 0.629, respectively. Both loci are in Hardy-Weinberg equilibrium, indicating that allele and genotype frequencies are stable in the population. The c.723C>T locus has a PIC of less than 0.25, which is a low polymorphism; the c.1144C>G locus has a PIC between 0.25 and 0.5, which is a moderate polymorphism (Table 2).

### 3.5. Association of Two Polymorphic Loci of the SLC11A1 with SCS in Chinese Holstein Cows

As shown in Table 3, for the c.723C>T polymorphic locus, the mean value of SCS of the *AA* type was significantly lower than that of the *AB* type (*p* < 0.05), and *AA* was the favorable genotype and *AB* was the unfavorable genotype for mastitis resistance. For the c.1144C>G polymorphic locus, *CC*, *CD*, and *DD* genotypes, the SCS means were not significantly different (*p* > 0.05).

### 3.6. Bioinformatics Analysis of Chinese Holstein Cows SLC11A1

**Figure 4:**
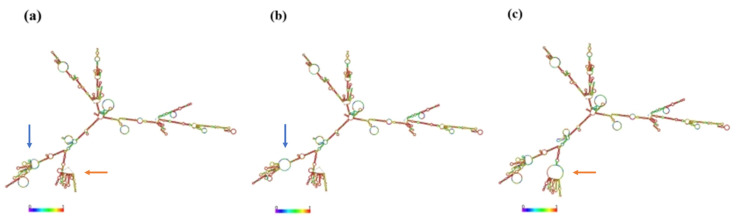
Predicted protein secondary structure before and after the SLC11A1 c. 723C>T and c. 1144C>G mutation. (**a**) The secondary structure of wild-type SLC11A1; (**b**) The secondary structure of mutate-type c. 723C>T. (**c**) The secondary structure of mutate-type c. 1144C>G. Note: The different colors represented the different base-pairing probabilities, where red > green > blue.

**Figure 5:**
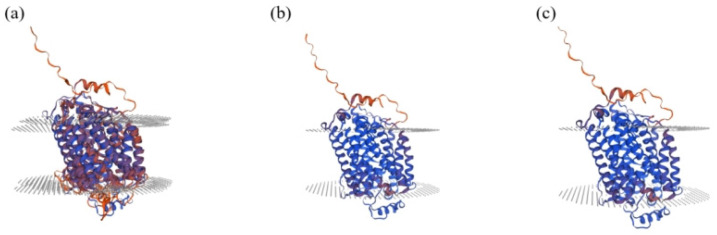
Predicted protein tertiary structure before and after SLC11A1 c. 723C>T and c. 1144C>G mutation. (**a**) The tertiary structure of wild-type SLC11A1; (**b**) The tertiary structure of mutate-type c.723C>T. (**c**) The tertiary structure of mutate-type c. 1144C>G.

Prediction of the secondary structure of *SLC11A1* mRNA from Chinese Holstein cows showed that the c.723C>T mutation and the c.1144C>G mutation resulted in changes in the secondary structure of the mRNA, and the minimum structural free energy increased from −770.17 kJ/mol to −768.67 kJ/mol and −768.57 kJ/mol, respectively, which decreased the stability of the mRNA secondary structure (Figure 4). It was predicted that the secondary structure of the proteins before and after the mutation would change slightly. 52.92% of the secondary structure of the proteins before the mutation was alpha helix, 12.04% was extended strand, 3.65% was beta turn and 31.39% was random coil. After the c.723C>T mutation, 53.47% was alpha helix, 12.59% was extended strands, 3.83% was beta turn and 30.11% was random coil. After the c.1144C>G mutation, 52.92% was alpha helix, 10.95% was extended strands, 3.65% was beta turn and 32.48% was random coil. The tertiary structure of the SLC11A1 protein also changed before and after the mutation (Figure 5).

### 3.7. The PPI Network of the SLC11A1 Protein

**Figure 6:**
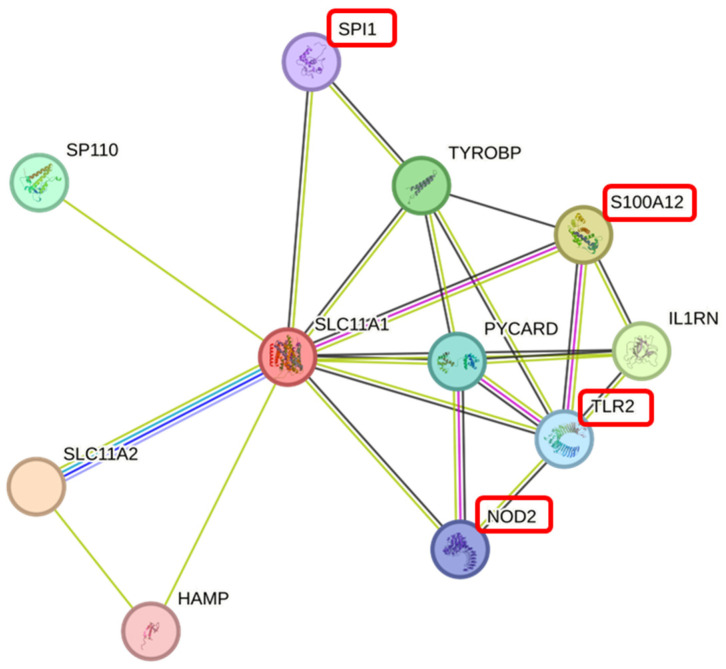
Protein networks that closely interact with SLC11A1 provided by the STRING database. Note: The proteins in the red boxes are strongly associated with mastitis resistance.

To further analyze the function of the SLC11A1 protein in Chinese Holstein cows, the network of target and related proteins was predicted using the STRING online tool. SLC11A1 was closely associated with the expression of genes involved in the regulation of the immune response and inflammation, with S100A12, NOD2, TLR2 and SPI1 being closely associated with mastitis resistance (Figure 6).

## 4. Discussion

Mastitis poses a significant challenge to the dairy industry, leading to considerable economic losses due to decreased milk yield and quality. It also has a substantial impact on the health and reproductive performance of dairy cows. Furthermore, mastitis is critical to maintaining public health [30,31,32]. The *SLC11A1* gene, previously defined as the Natural Resistance Associated Macrophage Protein 1 gene (*NRAMP1*), encodes an iron/divalent cation transmembrane protein located in phagocytosed lysosomal late membranes, particularly in macrophages [33]. *SLC11A1* plays a critical role in innate immunity [34] and is also important for macrophage recycling of metals (including iron) and digestion of apoptotic cells and aged erythrocytes [35,36]. In addition, microsatellite polymorphisms in the 3′ untranslated region (UTR) of the *SLC11A1* have been reported to confer protection against *Brucella abortus* [23,24,37], Mycobacterium Bovis [20,38], and *Mycobacterium avium* ssp. Paratuberculosis [39]. Moreover, the gene has been reported to be associated with natural resistance to mastitis in cattle [26].

In our study, we identified two mutation sites in the *SLC11A1* gene: c.723C>T and c.1144C>G. The c.723C>T locus contains two genotypes, of which type *AA* is in the majority and type *AB* is in the minority. The c.1144C>G locus contains three genotypes, of which type *CC* is in the majority and type *CD* and *DD* are in the minority. This may be due to the elimination of another pure type, possibly a recessive disease-causing gene, over a long period of time during the selection process. In addition, the PIC value of the c.1144C>G locus showed moderate polymorphism, suggesting that it could be used for breeding selection. Furthermore, both loci were in Hardy-Weinberg equilibrium, indicating that allele and genotype frequencies are stable in the population. This study also determined the association of c.723C>T locus and c.1144C>G locus with SCS in Chinese Holstein cows. Notably, a significant correlation between the c.723C>T locus and the mean value of SCS. The mean value of SCS of type *AA* was significantly lower than that of type *AB* at the c.723C>T polymorphic locus. These findings suggest that the c.723C>T locus may be a candidate gene associated with mastitis resistance. At the c.1144C>G locus, there was a difference between the SCS of the different genotypes, but it was not significant. Our findings are consistent with those of previous findings that have demonstrated the presence of a novel mutation at 1139 bp in exon 11 of the *SLC11A1* gene and a significant association of this SNP with clinical mastitis [26]. In addition, the SNP in the *SLC11A1* gene was identified as an important genetic marker and susceptibility factor for mastitis tolerance/susceptibility in Holstein and Swiss Brown cows [25]. It is suggested that *SLC11A1* may be a candidate gene associated with mastitis resistance.

Meanwhile, in the SNP site analysis, we found that the c.723C>T site and the c.1144C>G site had alanine replaced by valine and proline replaced by alanine, respectively. These mutations are closely related to the function and stability of the SLC11A1 protein. It has been shown that mutations in SNPs lead to changes in the secondary structure of mRNAs and the tertiary structure of proteins, which in turn affects animal phenotypic traits [40,41]. To elucidate the effects of these sense mutations on the function of the SLC11A1 protein, we performed a series of bioinformatic analyses. The results of secondary structure prediction showed that the minimum free energy of the c.723C>T and c.1144C>G sites was increased compared to that before the mutation, leading to a decrease in the stability of the secondary structure of the mRNA, which may affect the efficiency of gene expression. The c.723C>T and c.1144C>G sites alter the proportion of proteins with secondary structure to varying degrees, which may affect the stability of the protein’s secondary structure, thereby affecting the protein’s tertiary structure and causing changes in protein function. Therefore, changes in the structure of *SLC11A1* may influence mastitis resistance traits in Holstein cows.

However, the indirect role of *SLC11A1* in mastitis needs to be further investigated. Most physiological processes are supported by multiple proteins, so building protein interaction networks can improve our understanding of complex traits such as mastitis. We predicted a protein interaction network involving SLC11A1 interacting with 10 proteins, of which SPI1, NOD2, TLR2 and S100A12 have been reported as candidate genes associated with mastitis resistance. *SPI1* is an important member of the ETS transcription factor family and plays a key role in several steps of the inflammatory pathway. The *SPI1* gene was found to be a candidate for immunity and mammary gland inflammation in dairy cows [6]. *NOD2* is a key mediator of the innate and adaptive immune response to microbial infection. Association analysis of polymorphic loci of the *NOD2* with mastitis in dairy cows showed significant differences in the allele frequencies of the polymorphic loci [42,43]. *TLR2* plays a critical role in bacterial recognition and the host immune response during infection. *TLR2* is not only associated with inflammation and immunity in dairy mammary tissue [44], but also plays a key role in apoptosis and angiogenesis in mammary tissue [45]. S100A12 protein triggers a signal transduction cascade response leading to activation of the transcriptional co-regulator NF-KB and subsequent expression of pro-inflammatory cytokines. *S100A12* has been shown to enhance the antimicrobial capacity of dairy cows, helping to clear mammary tissue of infection [46], and can be used as a diagnostic indicator of subclinical mastitis in dairy cows [47]. These studies suggested that the *SLC11A1* was at the core of candidate regulatory genes for mastitis and that the expression of this gene potentially influences mastitis production. Therefore, it was important to analyze the association of *SLC11A1* polymorphisms with somatic cell scores.

## 5. Conclusions

In this study, we identified two mutations c.723C>T and c.1144C>G in the coding region of *SLC11A1* in Chinese Holstein cows. Association analysis showed that the c.723C>T locus of the *SLC11A1* gene had a significant effect on SCS. Meanwhile, at the c.723C>T and the c.1144C>G sites, alanine is replaced by valine and proline by alanine, respectively, and both mutations cause changes in the secondary and tertiary structure of the protein. Thus, *SLC11A1* may be a candidate genes associated with mastitis resistance. This study provided a theoretical basis for resolving the important role of *SLC11A1* in mastitis in dairy cows. This study focused on only two specific mutation sites in the gene; there may be other polymorphisms that also affect mastitis resistance, and functional experiments are needed to directly confirm the effects of these genetic variants on the expression and function of the gene. Future studies need to increase the sample size and further analyze the function of the two identified SNPs.

## Figures and Tables

**Table 1:** Primer sequence, product size and annealing temperature of *SLC11A1* in Holstein cows.

Gene	Primer	Primer Sequence (5′→3′)	Amplified Region	Size (bp)	Tm (°C)
*SLC11A1*	P1	F: TGTGAGGACAGCTGAGGAT R: CTGGAGAAGGGAATGGCTAT	Exons 1,2	1318	66
	P2	F: ACCGGGCTCCTCTGTCCATG R: GTGGCAAAGCTGGGGGTCTC	Exons 3	798	63
	P3	F: TATATGGCTCCCCTCTCCCT R: GTTACCGCAATGAATCCTCC	Intron 6	400	63
	P4	F: GGCTGACGGCCTCTCCCTAA R: TTCCTGTCCAGGGGCACCA	Exons 4	176	66
	P5	F: AGCCTTGCTGACGGGACTAC R: AAGAACTAGGGCCCCTCTG	Exons 5	165	58
	P6	F: CTGACCCAGGCCACTCTGGT R: TGAGAGGCACACACCCACG	Exons 6	114	65
	P7	F: TGCTCTCACCCTAGGGTTGC R: GGCCCCTGTTATGGCTCTGG	Exons 7	110	62
	P8	F: GGGACTTTCACTCACCCCAC R: CCTCCCCTCCAGTCTGCTCA	Exons 8	199	61
	P9	F: TCGATGCTTCCGTCCTTTTA R: CTTTGGAGTCCCCTGCAGAA	Exons 9	242	57
	P10	F: CGGTGCCCGCAGTTCAACAT R: GGAAGGAGGGGTGTGGTCG	Exons 10	241	62
	P11	F: TACAGGGGCGGAGCCTGCT R: CACATCCGAGTCCTGAGTGG	Exons 11	243	64
	P12	F: CGTCCCCTGGAACCCCAGTC R: GTTCCTGTTTGGCCCCACAG	Exons 12	259	65
	P13	F: TAACGGCAGGGTCCTTCAGC R: AGTGCGATAAGCCCAGTGCT	Exons 13	156	65
	P14	F: GCAGCCGATCCTGAATCTCC R: TGGTCTCCTCTCCTTGCCTC	Exons 14	243	60
	P15	F: CGGGGTTCCTCGTAGGTCT R: CCCTTGTCTGGCAGGCCAGT	Exons 15	353	65
	P16	F: ACGTGAGTTCCAGAGGGAC R: GGGGAAGCAAAGAATCTGC	3′ untranslated region	432	64

**Table 2:** Population genetic analysis of SNPs loci.

Polymorphic Site	Genotype	Genotype Frequency	Allele	Allele Frequency	Heterozy- Gosity	Effective Number of Alleles	PIC	χ2(*P*)
c.723C>T	AA (166)	0.790	A	0.895	0.188	1.231	0.170	2.88 (*p* = 0.09)
	AB (44)	0.210	B	0.105				
c. 1144C>G	CC (132)	0.629	C	0.805	0.314	1.458	0.265	3.09 (*p* = 0.08)
	CD (74)	0.352	D	0.195				
	DD (4)	0.019						

Note: PIC: polymorphism information content; PIC > 0.5 is high polymorphism, 0.25  ≤  PIC ≤ 0.5 is moderate polymorphism, PIC < 0.25 is low polymorphism; *p* > 0.05 is in Hardy-Weinberg equilibrium; *p* < 0.05 is not in Hardy-Weinberg equilibrium.

**Table 3:** Least squares means and standard errors for somatic cell scores of different genotypes.

Gene	Polymorphic Site	Genotype	Number of Samples	Somatic Cell Score
*SLC11A1*	c. 723C>T	AA	166	4.12 ^b^ ± 0.15
		AB	44	4.75 ^a^ ± 0.24
	c. 1144C>G	CC	132	4.19 ^a^ ± 0.17
		CD	74	4.34 ^a^ ± 0.20
		DD	4	4.65 ^a^ ± 0.28

Note: Least squares means with the same superscript for the same site have no significant difference (*p* > 0.05). Least squares means with the different superscripts for the same site differ significantly (*p* < 0.05).

## Data Availability

No new data were created or analyzed in this study. Data sharing is not applicable to this article.
